# Ellagic Acid-Derived Urolithins as Modulators of Oxidative Stress

**DOI:** 10.1155/2020/5194508

**Published:** 2020-07-28

**Authors:** Jasmina Djedjibegovic, Aleksandra Marjanovic, Emiliano Panieri, Luciano Saso

**Affiliations:** ^1^Faculty of Pharmacy, University of Sarajevo, Zmaja od Bosne 8, 71000 Sarajevo, Bosnia and Herzegovina; ^2^Department of Physiology and Pharmacology “Vittorio Erspamer”, Sapienza University, P.le Aldo Moro 5, 00185 Rome, Italy

## Abstract

Oxidative stress is a state of excess of prooxidative species relative to the antioxidant defenses (enzymatic and nonenzymatic) in a living organism. The consequence of this imbalance is damage of the major cellular macromolecules (carbohydrates, lipids, proteins, and DNA), which further leads to a gradual loss of tissue and organ function. It has been shown that oxidative stress plays an important role in the pathogenesis of many chronic diseases (cardiovascular, metabolic, and neurodegenerative diseases and cancer) and in the process of aging. Thus, many strategies to combat oxidative stress have been proposed and tested. In this context, food rich in antioxidants has received great attention. Pomegranate, berries, and walnuts have been recognized as “superfood” particularly for their cardioprotective effects. The common characteristic of these foods is the high content of ellagitannins. Since tannins are not bioavailable, they have been neglected in nutrition science and even considered antinutrients for a long time. However, this view has changed dramatically once it was recognized that ellagic acid, released from ellagitannins in the gastrointestinal system, is further metabolized by colonic microbiota to bioavailable compounds—known as urolithins. Thus, urolithins (3,4-benzocoumarin derivatives) have emerged as novel natural bioactive compounds and are now the focus of extensive investigations. So far, urolithins were shown to be powerful modulators of oxidative stress and agents with potential anti-inflammatory, antiproliferative, and antiaging properties. Furthermore, a few synthetic derivatives of urolithins were recognized as lead compounds for new drug development. Available data on urolithin synthesis, physicochemical and pharmacokinetic characteristics, biological activity, and safety will be presented in this review.

## 1. Introduction

Urolithins are a subgroup of dibenzo[b,d]pyran-6-ones (also named 3,4-benzocoumarins or dibenzo-*α*-pyrones) which were first isolated from natural sources (beaver scent glands) in 1949 [1]. Thereafter, their presence has been confirmed in many microorganisms, plants, human feces, and animal waste [2, 3]. Dibenzo[b,d]pyran-6-ones are biosynthesized by microorganisms, mostly by fungi through the polyketide pathway. Their presence in plants and animal and human intestines is probably a result of bacterial metabolism of ellagic acid [3]. Various dibenzo[b,d]pyran-6-ones detected in extracts of medicinal plants were described by Y. L. Garazd and M. M. Garazd [4], while those produced by fungi were described by Mao et al. [3]. Some of these compounds are toxic to humans and animals (mycotoxins), while the others show a number of beneficial health effects (antioxidant, antiallergic, antimicrobial, anti-inflammatory, antiproliferative, etc.) [3, 4].

**Figure 1:**
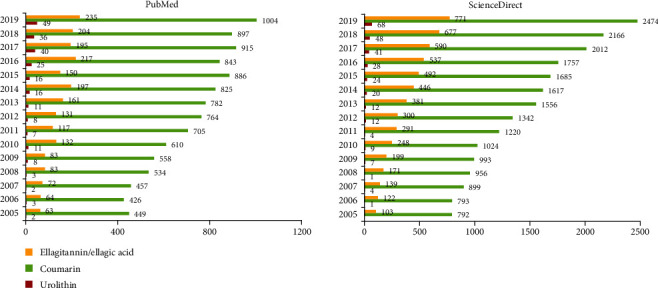
Timeline of published papers (number per year) corresponding to the keywords “urolithin”, “ellagitannins OR ‘ellagic acid'”, and “coumarin”.

In contrast to their structural homolog coumarin, the research on urolithins (and their natural precursor ellagic acid) has only started in the previous decade (Figure 1). Yet, the data collected so far are promising and support further research in this field. The focus of this review is ellagic acid-derived urolithins as bioactive compounds. Urolithins are attractive both as nutraceuticals and as intermediates in the development of novel pharmacological compounds (drugs). Data on their (bio) synthesis, pharmacokinetics, biological activity in the modulation of oxidative stress, and safety will be presented in the present review.

## 2. Urolithin Production by the Gut Microbiome

**Figure 2:**
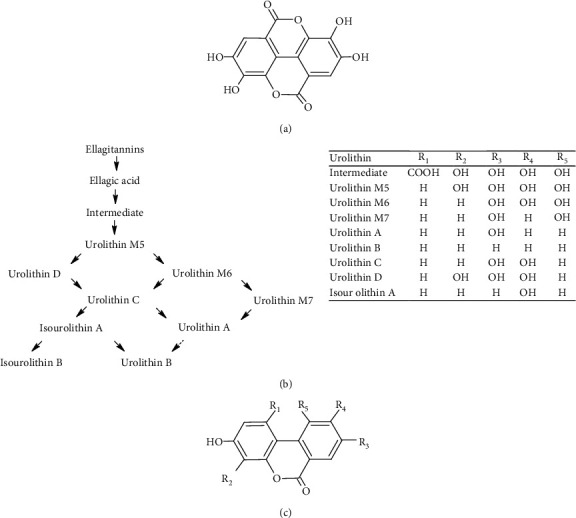
(a) Structure of ellagic acid, (b) diagram of its metabolism by the gut microbiota, and (c) structures of various urolithins.

Ellagic acid, mainly in the form of ellagitannins, is present in various medicinal and edible plants. High concentrations found in berries, pomegranate, and nuts make these foods good dietary sources [5]. During digestion, ellagitannins are converted to ellagic acid which is further metabolized by the gut microbiota to various dibenzo[b,d]pyran-6-one derivatives, named urolithins [6]. After cleavage and decarboxylation of one lactone ring in the molecule of ellagic acid, further metabolism occurs through a series of dehydroxylation reactions (Figure 2).

Contrary to poorly bioavailable ellagic acid, urolithins are more easily absorbed and have been detected in the circulation (mainly as glucuronide, sulfate, or methyl derivatives) at high nM to low *μ*M concentrations [7]. It is believed that urolithins are responsible for the many health effects of ellagic acid, ellagitannins, and food rich in these compounds. Urolithin presence in plasma and urine persists for a couple of days after oral intake, probably due to enterohepatic recirculation [8, 9]. The main isomers found in human biological fluids and tissues after ingestion of ellagitannin-rich food are urolithin A and urolithin B. A novel metabolite was recently identified in human feces, although the study was conducted on a limited number of participants [10].

Urolithin production in the intestine is dependent on the host microbiome, and three distinct metabotypes have been identified: A (producing only urolithin A), B (producing urolithin A, urolithin B, and isourolithin A), and 0 (only negligible urolithin production). These metabotypes seem to be quite stable and independent of food source, age, and health status [11]. The recognition of metabotype is essential in dietary intervention studies, but it also suggests that the exact amount of urolithins produced and absorbed from various dietary sources might be hardly estimated, especially when considering the effect of a dietary matrix, ellagitannin structures, and its variable food concentrations. In this regard, direct urolithin consumption as a dietary supplement might be a promising approach.

## 3. Urolithin Synthesis and Physicochemical Characteristics

Besides their biological activities, urolithins are also important intermediates in the synthesis of various dibenzopyranone derivatives with potential pharmaceutical use. For example, 4-bromo urolithin A was recently shown to be a very potent casein kinase 2 inhibitor [12]. On the other hand, urolithin B derivatives showed remarkable cholinesterase inhibitory activity which was comparable to that of galantamine and higher than that of rivastigmine [13]. Therefore, methods for efficient synthesis of these compounds are intensively developing.

**Figure 3:**
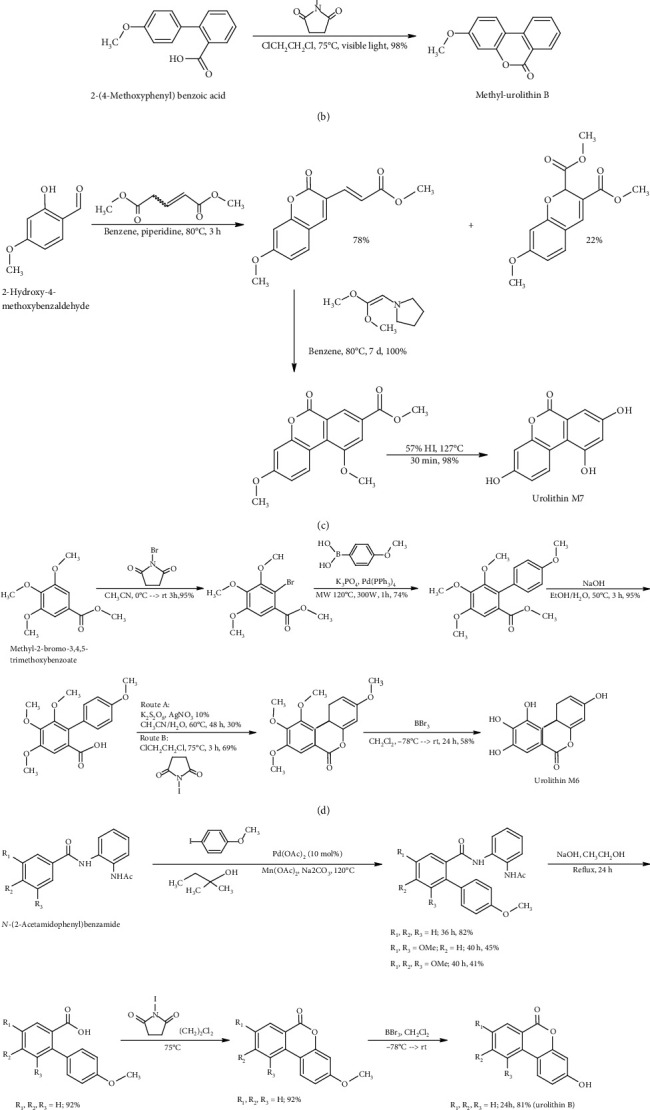
Strategies in urolithin synthesis: (a) from References [16, 17], (b) from Reference [18], (c) from Reference [19], (d) from Reference [20], and (e) from Reference [21].

Different strategies for urolithin synthesis were reported in the literature (e.g., copper-catalyzed Hurtley reaction, ellagic acid decarboxylative hydrolysis, inverse electron demand Diels–Alder reaction, and 2-bromobenzoic acid esterification followed by the Heck coupling) [14, 15]. Examples of urolithin synthesis are shown in Figure 3.

Urolithins have a molecular mass of less than 300 g/mol (e.g., 276.2 g/mol for urolithin M5 and 212.2 for urolithin B). The log *p* value is 2.7 for monohydroxy urolithin B, 1.1 for urolithin B glucuronide, and 1.3 for pentahydroxy urolithin M5. The number of hydrogen bond acceptors is typically less than 10 (e.g., 3 in urolithin B and 9 in its glucuronide), and the number of hydrogen bond donors is ≤5 (1 in urolithin B, 4 in urolithin B glucuronide, and 5 in urolithin M). Thus, these molecules do not show violations of Lipinski's “rule of five” [22, 23]. Furthermore, urolithins have 0 rotatable bonds (up to 3 when glucuronidated), and the polar surface area is typically <130 Å^2^ (143 Å^2^ for urolithin B glucuronide). Veber's rule states that a compound with ≤10 rotatable bonds and polar surface area ≤ 140 Å^2^ (or 12 or fewer hydrogen bond donors and acceptors) most likely will have a good oral bioavailability [24]. Another set of rules to be used in assessing “drug-likeness” of a certain compound were described by Ghose and coworkers and thus are commonly known as the Ghose filter. This filter defines the following “drug-likeness” constraints: log *p* is between -0.4 and 5.6, molecular weight ranges from 160 to 480, molar refractivity ranges from 40 to 130, and the total number of atoms is 20 to 70 [25]. The molar refractivity values of urolithin B, urolithin B glucuronide, and urolithin M5 are 58.3, 94.75, and 64.9, respectively. The total number of atoms in a molecule is between 20 and 70 for both urolithins and their glucuronides. Urolithins were recently assessed through the SwissADME web tool which provides researchers with a pool of predictive models for physicochemical properties, as well as pharmacokinetics, and drug-likeness [26]. The analysis showed high gastrointestinal absorption for urolithin B, but low absorption for urolithin M and urolithin B glucuronide. The software also predicted that urolithin B can cross the blood-brain barrier and act as the CYP1A2 inhibitor, in contrast with the urolithin B glucuronide or the highly hydroxylated urolithin M5. Moreover, none of these three substances appeared to be a substrate for P-glycoprotein (P-gp) which is normally responsible for the extrusion of the intracellular xenobiotics and thereby a factor limiting the efficacy of some drugs and other bioactive compounds. Still, it must be noted that some *in vitro* results suggest that P-gp and other ABC transporters (MRP, ABCG2/BCRP) might play a role in the urolithin transport and metabolism in certain cell lines (HT-29, MDCKII) [27]. In a quite recent study, urolithin B was detected in the brain of rats upon intravenous administration, confirming that this compound can cross the blood brain barrier, consistently with the software prediction [28].

## 4. Urolithin Pharmacokinetic Characteristics and Safety

Considering direct urolithin consumption, the first issue that has to be addressed is their stability in the gastrointestinal system. Using an *in vitro* digestion model (a sequence of oral, gastric, and pancreatic digestion, followed by a 24 h fecal fermentation), Mena et al. showed that urolithin B was more stable than urolithin B glucuronide and urolithin A. The recovery (% of the initial quantity) at the end of the last (colonic) step was 47 ± 8% for urolithin B, 30 ± 4% for urolithin B glucuronide (recovered as urolithin B, indicating a complete deglucuronidation during colonic fermentation), and 16 ± 5% for urolithin A [29]. However, this study proved the bioaccessibility of orally administered urolithin isomers. Bioavailability (i.e., the actual absorption in the circulation) after direct urolithin administration was tested only for urolithin A in healthy elderly subjects. Dose-dependent increase in maximum plasma concentration (*C*_max_) and total exposure (AUC) was observed (dose range of 250-1000 mg). The highest plasma concentration was found for urolithin A glucuronide, followed by urolithin A sulfate, and the parent compound with the dose-dependent corresponding *C*_max_ in ranges 1500-3000 nM, 200-400 nM, and 4-7 nM, respectively. The time to maximum concentration (*T*_max_) was 6 hours for all three compounds after administration of two lower doses (250 and 500 mg), while it was somewhat longer for the highest dose (1000 mg). The biphasic kinetics was shown for metabolites, probably due to enterohepatic circulation. The half-life (*t*_1/2_) ranged from 17 to 22 hours for the parent compound and urolithin glucuronide, while it was slightly longer (25-58 hours) for urolithin A sulfate. All the three compounds were eliminated from circulation in 72-96 hours. During 4 weeks of administration, a dose-dependent increase was also observed in total urolithin A (parent compound + metabolites) steady-state concentrations ranging from about 400 ng/mL for the lowest dose to about 600 ng/mL for the highest dose. The authors reported no accumulation in plasma after multiple-dose as compared to single-dose pharmacokinetics. Urolithin A was detected in muscle tissue in a concentration of about 6 ng/g tissue 8 hours after an oral dose of 2000 mg, mostly as a parent compound with only traces of urolithin A glucuronide found in two out of six participants [30]. This is an important finding since tissue distribution and cell availability of many natural compounds are often an issue. As already mentioned, urolithin glucuronides (the main form in the circulation) are quite large molecules with a lot of polar groups and their transmembrane transport is expected to be low. Moreover, according to some authors, it is possible that phase II metabolism could hamper urolithin activity. However, novel findings indicate that tissue availability of urolithins could actually be higher than expected due to the *in situ* deglucuronidation process. Ávila-Gálvez et al. described tissue deglucuronidation of urolithin A glucuronide in response to lipopolysaccharide-induced systemic inflammation in male Sprague-Dawley rats [31]. The authors observed a dramatic and statistically significant decrease in the urolithin A glucuronide/urolithin A ratio in all the tested organs of treated rats vs. controls, including the small intestine, liver, kidney, bladder, spleen, and lung as well as the urine. However, further research is needed to elucidate the precise mechanisms underlying this process. Piwowarski et al. also tested the effect of the inflammation on urolithin glucuronides. Here, the glucuronides of urolithin A, isourolithin A, and urolithin B isolated from the urine of a volunteer after ingestion of ellagitannin-rich food were shown to be cleaved by *β*-glucuronidase released from human neutrophils (upon *N*-formylmethionine-leucyl-phenylalanine stimulation), *Escherichia coli* standard strains, and clinical samples from patients with urinary tract infections [32]. Since *β*-glucuronidase is present at high concentrations in the sites of inflammation as well as in a tumor microenvironment, this could be one of the biologically relevant mechanisms underlying the protective effects of urolithins reported from many *in vitro* tests and suggested by epidemiological studies. Of note, some of the protective effects were also shown for glucuronide metabolites and not only for urolithin aglycone forms [14, 33]. On the other hand, it appears that urolithin metabolism could be cell-specific. A remarkable difference was reported from a study on the effects of urolithin aglycones (urolithin A, urolithin B, and isourolithin A) and their metabolites (sulfates and glucuronides) in two types of human breast carcinoma, MDA-MB-231 and MCF-7. In contrast to MDA-MB-231 cells, in MCF-7 cells, urolithins were metabolized much faster and mainly to sulfate, which did not exert any antiproliferative or estrogenic/antiestrogenic activity [34].

Regarding urolithins' safety upon direct consumption, we were able to identify only one toxicological study for urolithin A and none for the other isomers. The battery of genotoxicity assays (Ames test and *in vivo* and *in vitro* micronucleus assay, both with and without S9 metabolic activation) demonstrated that urolithin A is not genotoxic. After both oral and intravenous administrations to Wistar rats, the predominant metabolites were glucuronide and sulfate. After oral administration (1000 mg/kg bw), wide tissue distribution was found, although with the highest recovery in the gastrointestinal tract. The authors reported negative results for overall and organ toxicity after 28- and 90-day studies. The NOAEL was the highest dose applied (3451 mg/kg bw/day in males and 3826 mg/kg bw/day in females), corresponding to a human equivalent dose of approximately 557 mg/kg bw/day in males and 617 mg/kg bw/day in females [35].

In the urolithin A study in the elderly healthy volunteers, six adverse events were recorded in a single dose testing (2 in a placebo group of 6 participants and 4 in a test group of 18 participants). In a 28-day multiple ascending dose trial, 31 adverse effects were recorded in total (7 in a placebo group of 9 participants and 24 in a treatment group of 27 participants). All the adverse effects were mild to moderate and with one single exception were assessed to be unrelated or unlikely to be related to the product tested. There were no dropouts during the study and no reports of any clinical or laboratory abnormalities up to a dose of 2000 mg of urolithin A (the highest dose tested) [30].

Based mostly on the above presented findings, urolithin A already received a favorable review by the US FDA (Food and Drug Administration) under their generally recognized as safe (GRAS) notification program and can be used as a food ingredient at levels up to 1000 mg/serving, which is estimated to result in (applicant-reported) mean dietary exposure to urolithin A of 1183 mg/day in the consumers [36]. For comparison, the mean estimated dietary exposure to urolithin A (from ellagic acid in food) based on food consumption data from the 2013–2014 National Health and Nutrition Examination Survey reported by an applicant was 3.0 to 15.9 mg/day. The application for approval of urolithin A as a novel food was also submitted to EFSA (European Food Safety Authority) and is currently being processed. Although it is clear that urolithins are produced from ellagic acid in the human intestine, they are not known to be present in food and need to get approval before being used as nutritive additives or dietary supplements.

## 5. Urolithin Activity in the Modulation of Oxidative Stress

Oxidative stress is a state of excess of prooxidative species (primarily reactive oxygen species (ROS) and reactive nitrogen species (NOS)) relative to the antioxidant defenses (enzymatic and nonenzymatic) in a living cell or an organism. The consequence of this imbalance is damage to a variable extent to the major cellular macromolecules (carbohydrates, lipids, proteins, and DNA), which further leads to a gradual loss of tissue and organ function. It has been shown that oxidative stress plays an important role in the pathogenesis of many chronic diseases (cardiovascular, metabolic, and neurodegenerative diseases and cancer) and in the process of aging [37, 38]. Thus, various natural sources (food and medicinal plants) are tested for their ability to combat oxidative stress. In that respect, the identification of bioactive compounds and their mechanism of action is crucially important. Besides a direct antioxidative effect via radical scavenging, which can be easily measured *in vitro* but often fails to be clinically relevant, oxidative stress modulators can also modulate the activity of different cellular pathways that control the redox homeostasis *in vivo* [39, 40]. Of note, ellagic acid-derived urolithins were recently recognized as emerging oxidative stress modulators.

In order to comprehensively evaluate the antioxidative potency of seven different urolithins, Bialonska et al. applied a cellular assay which allows estimation of the cellular bioavailability of the test compounds and enables the detection of their antioxidative activity. Antioxidant activity in myelomonocytic HL-60 cells treated with phorbol-12-myristate-13 acetate (PMA) was determined by the DCFH-DA (2′,7′-dichlorodihydrofluorescein diacetate) method. The test measures the ability of the investigated compounds (urolithin A; urolithin B; urolithin C; urolithin D; 8-O-methylurolithin A; 8,9-di-O-methylurolithin C; and 8,9-di-O-methylurolithin D) to inhibit the DCFH oxidation by the generated ROS. The results of this study revealed that the antioxidative activity of urolithins was correlated with the number of OH groups and lipophilicity of the molecules. The highest antioxidant activity was detected for urolithin C (IC_50_ = 0.16 *μ*M) and urolithin D (IC_50_ = 0.33 *μ*M). Urolithin A showed less significant antioxidant activity (IC_50_ = 13.6 *μ*M), while urolithin B and all the methylated urolithins did not show antioxidant activity at all. The overall conclusion of this study was that urolithins may account for systemic antioxidant effects [17].

So far, dual redox nature of plant-derived polyphenols is widely known, making it necessary to investigate not only the antioxidant but also the prooxidant capacities of these compounds, since their physiological actions may depend upon their behavior as either an antioxidant or a prooxidant. Kallio et al. conducted for the first time a study to investigate the prooxidant properties of urolithins. Besides that, the aim was also to characterize the antioxidant properties of urolithins A and B using the *in vitro* ORAC assay, cell-based assays, and electrochemistry. In addition to redox properties, potential antiproliferative effects on HepG2 cells were also investigated. The ORAC values for ellagic acid (4.35 Trolox equivalent), urolithin A (6.67 Trolox equivalent), and urolithin B (5.77 Trolox equivalent) were two times lower than ORAC values for quercetin. The ratios between the ORAC values for urolithins and ellagic acid were 1.53 for urolithin A and 1.33 for urolithin B, so these ellagic acid metabolites can be described as relatively strong antioxidants. In a cell-based antioxidant activity assay on the promyelocytic cell line HL-60, wherein a reporter dye is oxidized by peroxyl radicals, urolithin A showed antioxidative action, while urolithin B did not show any obvious concentration-dependent antioxidant or prooxidant activity in the cell cultured medium. However, when the intracellular antioxidant activity was assessed, urolithins A and B showed no antioxidant properties, while instead they acted as prooxidants. In a copper-initiated prooxidant activity (CIPA) assay, both urolithins A and B were prooxidants, whereas urolithin A showed a stronger prooxidant activity. The authors further investigated the electrochemical oxidation of urolithins with cyclic voltammetry (CV) employing screen-printed carbon electrodes. The cyclic voltammograms of urolithin A showed both anodic and cathodic peaks at each scan rate. The cyclic voltammogram of urolithin B revealed an irreversible redox reaction as no cathodic peak was observed. Moreover, urolithins A and B caused significant decrease in the proliferation of HepG2 cells after 48 h incubation, while ellagic acid did not induce any changes in cell proliferation [41].

Mazumder et al. made an *in silico* investigation on the inhibitory potential of the constituents of pomegranate juice on the antioxidant defense mechanism. They hypothesized that although polyphenols present in pomegranate juice may scavenge free radicals and exert antioxidant activity, different compounds may interfere with enzymes involved in the antioxidant defense mechanisms and thereby paradoxically contribute to oxidative stress. This study was conducted to elucidate the potential of different constituents of pomegranate juice and their metabolites in affecting the cellular antioxidant defense system, using computational modeling analysis. Results showed that among others, urolithin A, urolithin A glucuronide, and urolithin B have the potential to inhibit catalase, SOD (superoxide dismutase), GR (glutathione reductase), GPx (glutathione peroxidase), and GST (glutathione-S-transferase), by interfering with their active catalytic sites. These findings suggest that these compounds can act both as prooxidants and as antioxidants [42].

The phosphatidylinositol-3-kinase (PI3K)/Akt pathway is responsible for many important cellular processes, including protein synthesis, proliferation, apoptosis, autophagy, glucose uptake, and metabolism. This pathway, among the other functions, mediates the metabolic effects of insulin at the cellular level. ROS at the same time activate PI3K and inactivate phosphatase and tensin homolog (PTEN), which is a negative regulator of Akt. Akt is activated through the phosphorylation at serine (Ser473) or threonine (Thr308) sites. Previously, it was reported that this activation is dependent on oxidative stress levels. Endothelial Akt-kinase plays a key role in the pathogenesis of cardiovascular complications in type 2 diabetes mellitus (T2DM), and therefore, the modulation of its activity would be of great physiopathological importance. Dirimanov and Högger conducted a study to identify subclasses of polyphenols, including urolithins, that can modulate the PI3K/Akt signaling pathway and thus be effective in the prevention and management of late T2DM complications. Here, the quantitative effects of the investigated compounds in endothelial cells *in vitro* were determined by ELISA and confirmed by Western blot analysis. Urolithin A showed significant and reproducible inhibition of Akt phosphorylation (35 ± 12%, *n* = 6, *p* = 0.001). Other urolithins (urolithins B, C, and D) did not show statistically significant inhibitory effects. Among urolithins, differences in inhibitory effects were recorded, most likely related to the different structures. Indeed, the presence of two OH groups at the C_3_ and C_8_ positions in urolithin A appeared to be important for the inhibitory effects [43]. Similar results were reported in two other studies of Komatsu et al. [44] and Piwowarski et al. [45] that were aimed at evaluating the anti-inflammatory potential of urolithins and their underlying mechanisms in lipopolysaccharide- (LPS-) stimulated murine RAW264 macrophages.

ROS are known proinflammatory mediators which can activate microglia and cause chronic neuroinflammation resulting in the onset of neurodegenerative diseases. Lee et al. investigated the antioxidant and anti-inflammatory effects of urolithin B in activated microglia. According to the results of Western blot analyses, urolithin B increased AMPK phosphorylation and decreased Akt, JNK, and ERK phosphorylation without affecting phospho-p38. These findings suggest a possible mechanism of anti-inflammatory activity. Urolithin B also showed antioxidant effects by inhibiting intracellular ROS production in LPS- (lipopolysaccharides from *Escherichia coli* serotype 055:B5) stimulated BV2 microglial cells. The probable mechanism of antioxidative action appears to be the inhibition of NADPH oxidase subunits along with the upregulation of heme oxygenase-1 (HO-1) [46]. Similarly, Xu et al. in their study concluded that urolithins A and B inhibit LPS-induced inflammation of microglia through the inhibition of the NF-*κ*B, MAPK, and PI3K/Akt signaling pathways [47].

At both cellular and molecular levels, inflammation has an important role in initiating and accelerating osteoarthritis development. Ding et al. conducted a study to investigate the possible anti-inflammatory action of urolithin A related to the attenuation of IL-1*β*-induced degradation of collagen II and aggrecan and the decreased production of inflammatory mediators via the ERK, JNK, p38, and NF-*κ*B pathways in rat chondrocytes. Results showed that the MAPK and NF-*κ*B pathways were involved in the protective effects of urolithin A since this metabolite inhibited the phosphorylation of MAPK pathway members thus protecting chondrocytes against IL-*β*-induced inflammation injury. The overall conclusion of this study was that urolithin A is a promising possible therapeutic agent for the treatment of osteoarthritis [48]. Importantly, the results of this study confirmed those previously reported by Fu et al. [49].

In another study, González-Sarrías et al. showed that urolithins (Uro-A, Uro-B, Uro-C, and Uro-D) and urolithin glucuronide conjugates in blood exerted potent protection against H_2_O_2_-induced cell injury. Results of the study revealed that neither the different hydroxylation patterns of urolithins nor the phase II metabolism was critical for their neuroprotective effects. No differences were observed among all the urolithins' aglycone and glucuronide conjugates, except for the Uro-B. Pretreatment with Uro-A significantly decreased the percentage of late apoptosis (12%, *p* < 0.05) in human neuroblastoma SH-SY5Y cells, compared to H_2_O_2_ treatment alone. Urolithins reduced intracellular ROS levels, increased mitochondrial oxidation-reduction activity, and decreased apoptosis induced by oxidative stress, by preventing the caspase-3 activation. The authors also noted that these neuroprotective effects of urolithins were less pronounced than those obtained with ellagic acid under the same conditions [50]. However, in consideration of the already discussed issues regarding ellagic acid metabolism and absorption, it seems to be at least questionable that ellagic acid might reach the effective concentration in the brain tissue.

Similar findings on LPS-BV-2 microglial cells and human neuroblastoma SH-SY5Y cells were reported in another study by DaSilva et al. [51].

It is well known that redox status has a great impact on the development of neurodegenerative diseases. Therefore, Cásedas et al. investigated whether urolithin A could have antioxidative and neuroprotective effects on the murine Neuro-2a neuroblastoma cell line. Results showed that urolithin A had a great antioxidant capacity, as measured in the ORAC test (13.1 *μ*mol TE/mg) and exerted a number of cytoprotective effects. Indeed, this metabolite improved mitochondrial activity in cells exposed to H_2_O_2_, decreased lipid peroxidation in cells subjected to oxidative stress, enhanced the activity of superoxide dismutase (SOD), catalase (CAT), glutathione reductase (GR), and glutathione peroxidase (GPx), and dose-dependently increased the expression of peroxiredoxins 1 and 3 (Prx1 and Prx3) compared to control [52].

A study of Chen et al. tested the neuroprotective effect of urolithin A on H_2_O_2_-induced oxidative injury in PC12 (pheochromocytoma) cells. Results showed that urolithin A effectively prevented H_2_O_2_-induced apoptosis in PC12 cells by markedly regulating in the opposite ways the protein levels of caspase-3 and Bcl-2 (both *p* < 0.01). The *in vivo* D-galactose-induced brain aging model showed that urolithin A significantly suppressed the upregulation of miR-34a induced by D-galactose. The overall conclusion was that urolithin A may have neuroprotective effects, especially in preventing D-gal-induced brain aging through the activation of the miR-34a-mediated SIRT1/mTOR signaling pathway [53].

Later on, Zheng et al. conducted a study on possible protective effects and molecular mechanisms of urolithin B on myocardial ischemia/reperfusion (IR) injury. Here, treatment with urolithin B significantly decreased the levels of superoxide anion radicals and lipid peroxidation products in H9c2 cells, restoring also the SOD expression. To examine the potential role of autophagy in the protective effects of urolithin B, levels of LC3 and p62 were determined. IR increased the ratio of LC3II/I and conversely decreased p62 levels, which were significantly reversed by urolithin B treatment. Accumulation of p62 and its interaction with KEAP1 had an important role in the antioxidative effects mediated by urolithin B, promoting Nrf2 nuclear translocation and subsequent expression of antioxidant enzymes such as HO-1, GSTP1, and NQO1. Results suggested that protective effects of urolithin B could be partially due to the modulation of autophagy [54]. Additionally, Tang et al. revealed that urolithin A could reduce myocardial apoptosis following ischemia/reperfusion. The authors concluded that the protective effect of urolithin on cardiomyocyte apoptosis during hypoxia/reoxygenation injury was at least partially mediated by the activation of the PI3K/Akt signaling pathway [55].

Nonenzymatic protein glycation reactions are usually observed in conditions related to oxidative stress. As a result, advanced glycation end products (AGEs) are formed and can be accumulated mainly in proteins with a long half-life, altering their structural and functional properties. It seems that AGEs have a significant role in the progression of cardiovascular complications associated with diabetes and also Alzheimer's disease and neuropathy. In this regard, Verzelloni et al. investigated the inhibitory activity of urolithins A and B against AGE formation, using concentrations that can be reached *in vivo* and assessing their ability to counteract mild oxidative stress in cultured human neuron cells. They also evaluated iron chelation ability with a ferrozine assay and radical scavenging activity, which was measured with the ABTS (2,2′-azino-bis(3-ethylbenzothiazoline-6-sulfonic acid)) assay. The results showed that urolithins A and B at the lowest concentrations of 0.5 *μ*mol/L had weak antiglycative activity, while their activity was much stronger when higher concentrations (1 *μ*mol/L) were used. Urolithin A had substantial antiglycative activity at the highest concentration (10 *μ*mol/L). The antiglycative activity of urolithin B at the highest concentration (10 *μ*mol/L) was only slightly higher than that observed at 1 *μ*mol/L. Both urolithins showed much higher activity than all the other colonic polyphenol metabolites tested, including pyrogallol (raspberry/pomegranate “ellagitannin group”), colonic metabolites of “coffee group” polyphenols (dihydrocaffeic acid, dihydroferulic acid, and feruloylglycine), and “berry/red wine anthocyanin group” polyphenols (3-hydroxyphenylacetic acid, 3,4-dihydroxyphenylacetic acid, and 3-methoxy-4-hydroxyphenylacetic acid). In the ABTS assay, both of urolithins showed low activity, therefore suggesting that the antiglycative effect was not exclusively related to their antioxidative capacity. Neither of the urolithins was able to bind iron at 50 *μ*mol/L. Urolithin B showed stronger neuroprotective effects compared to urolithin A and all the other tested metabolites *in vitro*. Both urolithins showed protective effects with an increase in cellular viability after induction of oxidative stress with 2,3-dimethoxy-1,4-naphthoquinone (DMNQ) [56]. Kim et al. examined possible mechanisms of neuroprotective action of urolithin A against H_2_O_2_-induced oxidative stress *in vitro*. Here, after treatment with urolithin A, the expressions of cytochrome c, cleaved caspase-9, cleaved caspase-3, and cleaved poly (ADP-ribose) polymerase (PARP) were suppressed, confirming that urolithin A attenuated apoptotic cell death in H_2_O_2_-exposed SK-N-MC cells. In brief, urolithin A decreased ROS production in cells, inhibited the mitochondrial-related apoptosis pathway, and modulated the p-38 MAPK pathway [57].

Both of these studies were conducted in human neuroblastoma SK-N-MC cells, as reported by the authors. Although the SK-N-MC cell line is often used as a model for testing neuroprotective effects [58–61], it must be noted that this cell line is now widely regarded as having originated from Askin's tumor related to Ewing's sarcoma and not neuroblastoma as originally described [62].

Apart from reducing sugars, advanced glycation end products (AGEs) can also be formed in the reaction of nucleophilic groups of macromolecules with reactive carbonyl species (RCS). RCS such as glyoxal (GO), methylglyoxal (MGO), and 3-deoxyglucosone (3-DG) can be formed as side products of several enzymatic pathways involving carbohydrates. Biological effects caused by RCS seem somewhat similar to those induced by ROS. Methylglyoxal is one of the most reactive glycation agents, so it is a relevant target so its inhibition might be useful to prevent or at least attenuate the formation of AGEs. Liu et al. investigated the *in vitro* antiglycation effects of a standardized pomegranate fruit extract (PE) along with its major phenolic constituents, punicalagin (PA), ellagic acid (EA), and gallic acid (GA) as minor phenolic constituents as well as urolithins A and B. Inhibitory effects of the tested samples were evaluated with BSA-fructose and G.K. peptide-ribose assays, MALDI-TOF mass spectrometry, and circular dichroism (CD). Additionally, the dicarbonyl-scavenging properties of the samples were also examined by HPLC-DAD methods. Results showed that PA and EA were the major contributors to the antiglycation effects of PE, but urolithins A and B also showed promising antiglycation activity which was similar to that of the positive control. All the tested compounds showed dicarbonyl-scavenging capacity, but in the case of urolithins, it was weaker than that of the positive control [63].

Enhanced production of ROS is connected with peripheral inflammatory responses. In the study conducted by Ishimoto et al., *in vivo* anti-inflammatory and antioxidant activities of urolithin A were evaluated in the carrageenan-induced paw edema mouse model with an ORAC assay. The association between plasma ORAC scores and levels of urolithin A in plasma after oral administration in mice was investigated. Results indicated a strong correlation of plasma urolithin A levels and the plasma ORAC scores after oral administration of urolithin A. These findings can explain the profound anti-inflammatory effects exerted by urolithin A [64].

Subsequently, the study conducted by Saha et al. revealed that urolithin A potently inhibits heme peroxidases, namely, myeloperoxidase (MPO) and lactoperoxidase (LPO), compared to ellagic acid. Urolithin A significantly reduced the MPO and LPO activities in both dose- and time-dependent manners. Also, inhibition of MPO and LPO by urolithin A could be prevented by neutrophil gelatinase-associated lipocalin (NGAL), which is expressed in neutrophils and is involved in innate immunity by sequestrating iron, thus limiting bacterial growth. The Chrome Azurol S assay suggested that ellagic acid was capable of binding iron, whereas urolithin A failed to chelate iron. However, this allows urolithin A to retain its ability to inhibit peroxidase in the presence of ferric ion. As a consequence, urolithin A significantly reduced superoxide generation induced by phorbol myristate acetate (PMA) in neutrophils. These findings pointed out that urolithin A does inhibit not only MPO and LPO but also other prooxidant enzymes. To confirm these findings *in vitro*, urolithin A and ellagic acid were tested for their efficacy against PMA-induced ear edema in mice. Here, treatment with urolithin A was almost comparable to a positive control (indomethacin), which markedly reduced ear edema by 47.5%. Urolithin A also inhibited MPO activity, which suggests the potential role of urolithin A as an anti-inflammatory agent [16].

Singh et al. showed that urolithin A not only exerts the anti-inflammatory activity but also upregulates epithelial tight junction proteins through activation of AhR-Nrf2-dependent pathways. The anti-inflammatory activity was evidenced by a significant decrease in LPS-induced IL-6 and TNF-*α* both *in vitro* (mouse bone marrow-derived macrophages) and *in vivo* (LPS-induced peritonitis mouse model). While the anti-inflammatory effect was partially preserved in Nrf2^−/−^ cells, it seems to be strongly AhR-dependent. AhR-Nrf2 pathways are essential for urolithin A-induced upregulation of tight junction proteins. Urolithin A was shown to attenuate colitis in preclinical models as both prophylactic and therapeutic agents and could be an even better therapeutic option than anti-TNF-*α* antibodies currently used in the treatment of irritable bowel disease [65].

Endothelium-derived NO has a critical role in regulating vascular homeostasis, and its bioactivity is impaired in atherosclerosis and related diseases. Loss of bioactivity under these conditions is related to increased oxidative stress, especially caused by enhanced production of superoxide anion and accumulation of lipid peroxidation products. Therefore, antioxidant supplementation can restore endothelial vasomotor function. In this respect, Spigoni et al. assessed *in vitro* effects of urolithin A, urolithin B, and urolithin B glucuronide on endothelial function in primary human aortic endothelial cells (HAECs). The study was aimed at investigating effects of urolithins on the activation of endothelial nitric oxide synthase (eNOS) and the release of NO. Urolithins were tested both individually and as a mixture. This study was the first in assessing the effects of a mixture of urolithins at physiologically attainable concentrations on endothelial cell function. Results revealed that the mixture was more potent in releasing NO compared to individual urolithins. These findings suggest that benefits related to the ellagitannin-rich diet could be due to the combined activity of different metabolites [66].

Oxidized low-density lipoprotein (ox-LDL) can cause changes in various endothelial functions, impairing NO synthesis and stimulating endothelial cell expression of proinflammatory cytokines. This can lead to further inflammation, endothelial dysfunction, and atherogenesis. Han et al. investigated the effects of urolithin A on ox-LDL-induced endothelial dysfunction. Results showed that urolithin A in probable physiological concentrations could dose-dependently attenuate the ox-LDL-induced increase in LDH levels in human artery endothelial cells (HAECs). Moreover, urolithin A increased also the production of NO in a dose-dependent manner, suggesting that it might promote the NO synthesis by modulating the expression of endothelial nitric oxide synthase (eNOS) [67]. This is in agreement with the results obtained by Rosenblat et al. showing that both urolithins A and B significantly and dose-dependently inhibited copper ion-induced LDL oxidation, as measured by the TBARS or by the lipid peroxide assays [68].

Also, Larrosa et al. conducted a study to evaluate the effects of diet supplementation with a pomegranate extract (PE) and urolithin A on a dextran sodium sulfate- (DSS-) induced colon inflammation rat model to also assess whether the effects might be related to urolithins. Here, the antioxidant status of plasma was assessed with the ferric-reducing antioxidant power (FRAP) assay, while lipid peroxidation levels in colon tissue were evaluated by thiobarbituric acid-reactive substances (TBARS) measurement. Results showed that only the administration of PE ameliorated the antioxidant status in plasma, while urolithin A showed low antioxidative capacity in the FRAP assay. Peroxidation levels in rats supplemented with PE were significantly lower compared to control, while urolithin A administration did not produce any effect. Results also showed that both PE and urolithin A supplementations were able to abrogate the NO production by suppressing iNOS induction triggered by DSS treatment in rats. Overall, supplementation with urolithin A was more effective than that with PE in ameliorating the inflammation. The authors also noted that colonic inflammation could lower ellagic acid conversion to urolithins [69], while other work from Zhao et al. also suggested protective effects of urolithin A in high-fat diet-induced metabolic inflammation, based on the recovery of tissue damage in the colon and the regulation of the gut microbiota [70].

Olennikov et al. analyzed the commercial formulation known as Padma Liver regulator, which contains chebulic ellagitannins indicated for weak liver function and also recommended for preventing liver damage. One aim of the study was to estimate the hepatoprotective potentials of urolithins against tert-butyl hydroperoxide-induced experimental hepatocyte injury. The results suggested that urolithins in the presence of t-BHP protected the hepatocytes against the oxidative injury in a dose-dependent manner, acting as antioxidants. Indeed, the level of malondialdehyde (MDA) production in urolithin C-treated cells was lower than those in urolithin A and B groups demonstrating its good antioxidant properties. This is explained by the fact that urolithin C has the highest number of hydroxyl groups, while the least active was urolithin B having only one monohydroxy-substituted phenolic ring [71].

An early key step in the development of hepatocellular carcinoma is characterized by overexpression of proinflammatory molecules and the release of different growth factors. This is a result of ROS overproduction and metabolic alterations in the liver cancer cells. By using HepG2 cells as a model, Wang et al. explored the effects of urolithin A on the expression of NF-*κ*B-related inflammatory factor and ROS formation in H_2_O_2_-induced oxidative stress. The effects of urolithin A on ROS production and superoxide dismutase (SOD), malondialdehyde (MDA), and glutathione peroxidase (GSH-Px) levels were evaluated in response to H_2_O_2_ treatment. Here, the incubation of HepG2 cells with urolithin A led to the inhibition of ROS generation by nearly 50%, reinforcing the levels of SOD and GSH-Px, while in untreated cells, a significant increase in MDA was observed. The authors proposed that one potential mechanism of urolithin A action in HepG2 cells might derive from the decreased release of proinflammatory mediators caused by the suppression of the NF-*κ*B signaling pathway, followed by inhibition of oxidative stress [72].

ROS have the potential to cause cellular damage, especially to DNA, RNA, lipids, and proteins, that can eventually promote carcinogenesis. Kojadinovic et al. tried to characterize the intracellular response to oxidative stress after treatment of the human adenocarcinoma Caco-2 cell line with physiological amounts of urolithins mixture (urolithins A, B, C, and D). To determine the protective effect of urolithins, two types of treatments were used: a long-term preincubation condition and a short-term incubation treatment. For all the measurements in tested cells, oxidative stress was induced with H_2_O_2_ and cells were pretreated with urolithin mixture in a concentration of 30 *μ*M. The mixture of urolithins reduced the generation of ROS in both the experimental conditions (short term and long term), despite that a more pronounced effect was observed in the short term. Effects of urolithins were also evaluated by measuring the activity of specific antioxidant enzymes, such as superoxide dismutase (SOD), catalase (CAT), and glutathione peroxidase (GSH-Px) in Caco-2 cells. The addition of urolithins in growth medium significantly decreased the activity of CAT (*p* < 0.05), while SOD and GSH-Px activity was unaffected. These data indicate that the mixture of urolithins can attenuate the induced oxidative stress in the Caco-2 cells. Even short-term treatment can cause a decrease in ROS formation in cells, preventing the oxidative damage. Additionally, urolithins can modulate the activity of antioxidative enzymes [73].

The mitogen-activated protein kinase (MAPK) cascades, consisting of the extracellular signal-related kinases (ERK1/2), the c-Jun N-terminal kinases (JNK), the p38 kinase (p38), and the big MAP kinase 1 (BMK1/ERK5) pathway, play an important role in various cellular processes such as cell growth, differentiation, development, cell cycle, survival, and cell death. The p38 kinase is involved in malignant invasion and metastasis of bladder cancer. Qiu et al. conducted a study aimed at investigating the antiproliferative properties of urolithin A, urolithin B, 8-OMe-urolithin A, and ellagic acid in T24 human bladder cancer cells. They also evaluated the antioxidative activity of these compounds and their effects on caspase-3 activation and mRNA and/or protein of p38-MAPK (mRNA, phosphorylated protein), MEKK1 (mRNA, protein), c-Jun (mRNA, phosphorylated and nonphosphorylated protein), p53 (mRNA), cleaved caspase-3 (protein), and peroxisome proliferator-activated receptors gamma (PPAR-*γ*, protein). Oxidative stress induced by H_2_O_2_ in T24 human bladder cancer cells was estimated by measuring the intracellular content of ROS and MDA as well as the SOD activity. After incubation with different urolithins and ellagic acid, both the ROS and MDA levels were significantly decreased, whereas SOD activity markedly increased compared to the negative control. Results also suggested that urolithins and ellagic acid modulated both the transcription and protein content of p38-MAPK, MEKK1, and c-Jun in T24 cells, confirming possible antiproliferative effects on T24 cells [74].

One of the most widely diffused alterations across several types of malignant cancers is related to the functional inactivation of the oncosuppressor p53 that largely contributes to the acquirement of a chemoresistant phenotype [75, 76]. Thus, the reactivation of p53 represents an attractive therapeutic option in oncology that has been shown to promote tumor regression due to the induction of senescence in sarcomas and carcinomas or the activation of apoptosis in lymphomas [75, 77]. In case of p53 mutation or deletion (present in more than 50% of cancers), this reactivation could be achieved through gene therapy or targeted therapy aimed at substituting the p53 mutant form with the corresponding wild-type form. Of note, in tumors expressing the p53 wild-type form, its activity is often diminished by the overexpression of p53 inhibitors, namely, the E3 ubiquitin-protein ligase MDM2 (or MDM4) [75, 78]. In such tumors, the therapeutic strategy is aimed at disrupting the binding of p53 inhibitors to its target. Although still in its early phase, research on p53 reactivation in the context of cancer therapy is rapidly emerging. Besides some small molecules acting as MDM2 inhibitors already in the clinical trials, many natural compounds are currently tested for their p53 restoration activity in cancer cells. Several studies demonstrated such activity for urolithins. For instance, Giménez-Bastida et al. [79] reported that urolithin A applied in physiologically relevant noncytotoxic concentrations (0.5, 1, and/or 10 *μ*M) dose-dependently induced cellular senescence in p53 wild-type human colon cancer HCT-116 cells. This effect was not observed in two other cancer cell lines, namely, Caco-2 (p53-null) and HT-29 (mutant p53), as well as nontumorigenic CCD18-Co (wild-type p53) cells. On the other hand, urolithin B, urolithin C, isourolithin A, and ellagic acid did not exert this effect in the tested cell lines. In contrast to apoptosis, which can be induced at much higher concentrations, the induction of cellular senescence suggests the potential chemopreventive role of a regular dietary intake of urolithin precursors, at least for urolithin A-producing metabotypes. Another important point is the lack of effect resulting from testing with urolithin A metabolites (glucuronide or sulfate). This can also explain the absence of effect in Caco-2 and HT-29 cells, which metabolize urolithins at a much higher rate in comparison to HCT-116 cells.

Norden and Heiss also reported that urolithin A-induced p53 stabilization in HCT-116 cells not only exerted antiproliferative effects (IC_50_ = 19 *μ*M) but also synergized with the anticancer drug oxaliplatin. In the absence of p53, a significant decrease in urolithin A antiproliferative activity was however noted (IC_50_ = 38 *μ*M) [80].

Both p53-dependent and p53-independent antiproliferative effects of urolithin A were demonstrated in the three human prostate cancer lines with different p53 activities, namely, LNCaP (p53^+/+^), 22RV1 (p53^−/+^), and PC3 (p53^−/−^) cells. Here, an increased expression in both p53 and MDM2 was observed in LNCaP and 22RV1 cells, but this was paralleled by a strong inhibition of their reciprocal interaction. In contrast, in PC3 cells, urolithin A downregulated MDM2 and upregulated p21 and p14ARF expression in a p53-independent manner [78].

The role of p53 in cancer formation and progression involves the regulation of oxidative stress by controlling the expression of various antioxidant and metabolic genes. The final effect can be either antioxidant or prooxidant, depending on the extent of stress signals. Under the physiological conditions of low stress, p53 lowers ROS levels via induction of antioxidant genes (sestrins, TIGAR, GPX1, ALDH4, GLS2, and Parkin) and the stabilization of Nrf2. This antioxidant activity protects cells from oxidative stress-induced DNA damage and promotes cell survival. Under the conditions of severe stress, p53 induces prooxidant genes (PIG3, PIG6, FDRX, Bax, and Puma) thus increasing ROS levels in the cells. This increase in ROS levels further activates p53, leading to a high accumulation of ROS. The resulting state of oxidative stress leads to apoptosis and senescence, preventing the propagation of mutated cells. In p53-null cells, the elevation of intracellular ROS levels increases DNA oxidation and the rate of mutagenesis, and the use of antioxidants can reverse this effect [81]. In contrast to the wild-type p53, mutant p53 isoforms are not functional and support tumor progression. ROS formation is also enhanced by the Warburg effect, which was shown to be sustained in cells with mutant p53 proteins. However, mutant p53-driven ROS accumulation was also shown to enhance cell sensitivity to H_2_O_2_ treatment. It was then speculated that mutant p53-bearing cancer cells could be significantly more sensitive to prooxidant drugs [82]. In conclusion, the final effect of p53 activation seems to be dependent on the cell type, p53 isoform, level of p53 activity, and its location within the cell and can promote cell proliferation, migration, further genotoxic damage, senescence, or cell death.

Urolithin A was also shown to regulate mitochondrial function. The decline in mitochondrial function, characterized by changes in organelle morphology, insufficient ATP production, accumulation of mitochondrial DNA mutations, increased production of mitochondrial ROS, and oxidative damage to macromolecules, is one of the hallmarks of aging and age-related diseases. Since mitophagy enables the degradation of damaged mitochondria and it is significantly impaired in several human pathologies (neurodegenerative disorders, cardiovascular pathologies, and cancer), there is a rising interest in therapeutic interventions aimed at the induction of mitophagy. Ryu et al. found that urolithin A potently induces mitophagy and through this mechanism it extended lifespan and improved fitness during aging in *Caenorhabditis elegans* as well as muscle function and exercise capacity in rodents [83]. This corroborates the finding of Boakye et al. who demonstrated that urolithin A increased autophagic flux in murine J774.1 macrophages in a concentration-dependent manner, thereby contributing to anti-inflammatory action. An elevated autophagic flux could be attributed to suppression of LPS-induced phosphorylation of AKT and its downstream targets TSC2, mTOR, and p70S6K. The increased autophagic flux further contributed to the alleviation of LPS-stimulated proinflammatory M1 polarization in J774.1 macrophages (inhibition of iNOS/Cox2/pro-IL-1*β* expression and NO and ROS release). Finally, the urolithin A-induced increase in autophagic flux contributed to the reduced nuclear abundance of NF-*κ*B, presumably by interfering between the NF-*κ*B release from its complex with the inhibitor and its transport into the nucleus [84].

Elevated autophagy, augmented mitochondrial biogenesis, and attenuated NF-*κ*B activation were also recorded in the livers of C57BL/6 male mice fed a high-fat diet after intraperitoneal application of urolithin A in physiologically relevant concentrations (20 *μ*g/mice). Augmented mitochondrial mass and activity were demonstrated to contribute to the triglyceride- (TG-) lowering effect of urolithin A. This effect was probably due to a significant increase in the expression of mitochondrial genes related to beta-oxidation, namely, carnitine palmitoyltransferase 1 (Cpt1) and sirtuin 1 (Sirt1). Simultaneously, levels of the lipogenic gene and protein expression (e.g., fatty acid synthase and stearoyl-CoA desaturase 1) were decreased. Moreover, livers from urolithin A-treated mice had augmented expression of ROS-scavenging enzymes, such as SOD1 and SOD2. Furthermore, urolithin A decreases macrophage infiltration into adipose tissue and favors M2 over M1 polarization of macrophages. The overall observed systemic effects were reduced hepatic and adipose inflammation and improved insulin sensitivity in the dietary challenge with a high-fat diet [85].

The beneficial effects of urolithin A on mitochondrial health were also reported in the first human randomized, double-blind, placebo-controlled clinical study in sedentary elderly subjects. Although the study duration (4 weeks) did not allow the assessment of physiological effects (muscle function), it showed a global impact on the biomarkers of mitochondrial health following urolithin A oral administration at doses of 500 and 1000 mg. A dose-dependent decrease in acylcarnitine levels (C8 to C14 and >C20) was observed in plasma, without change in free carnitine levels, indicating that UA improves fatty acid oxidation at the level of the whole body. Mitochondrial abundance was evaluated by measuring the ratio of mitochondrial DNA to nuclear DNA, which tended to increase, albeit not significantly. Treatment with UA was seen to upregulate the transcription of mitochondrial genes. Taken together, these data demonstrate that urolithin A stimulated mitochondrial biogenesis in the skeletal muscle of the participants. Further studies of longer duration are needed to evaluate urolithin A effects on muscle strength as well as possible variations in a dose-effect relationship from chronic exposure [30].

## 6. Conclusions

Ellagic acid-derived urolithins are produced by the gut microbiome, absorbed, and regarded to be responsible for the systemic beneficial health effects associated with the consumption of ellagitannin-rich food. The research interest in urolithins has considerably grown in the past decade. Efficient methods for urolithin synthesis have been developed, many tests on their activity were performed, their bioaccessibility was proven in an *in vitro* digestive simulation test, and toxicological studies have shown a good safety profile for urolithin A. The results obtained so far confirm that urolithins can potentially be used to modulate oxidative stress and ameliorate tissue damage through different mechanisms. The first functional food containing urolithin A is already on the horizon. The evidence also indicates that urolithin metabolism could be cell-specific, which in turn alters their activity *in vitro*, a phenomenon that warrants further investigation. On the other hand, a couple of synthetic urolithin derivatives were shown to be potent and selective inhibitors of certain enzymes (CK, cholinesterase) which makes them novel drug candidates. In conclusion, urolithins are promising molecules with many potential therapeutic activities that are yet to be validated in clinical trials. It is anticipated that these compounds will be the focus of extensive research in the near future, hopefully providing new and exciting results in different physiopathological settings.
